# Computed Tomography Risk Disclosure in the Emergency Department: A Survey of Pediatric Emergency Medicine Fellowship Program Leaders

**DOI:** 10.5811/westjem.2018.4.36895

**Published:** 2018-06-04

**Authors:** Jennifer R. Marin, Karen E. Thomas, Angela M. Mills, Kathy Boutis

**Affiliations:** * University of Pittsburgh School of Medicine, Department of Pediatrics and Emergency Medicine, Pittsburgh, Pennsylvania; † University of Toronto, Hospital for Sick Children, Department of Diagnostic Imaging, Toronto, Ontario, Canada; ‡ Columbia University Vagelos College of Physicians and Surgeons, Department of Emergency Medicine, New York, NY; § University of Toronto, Hospital for Sick Children, Division of Emergency Medicine, Toronto, Ontario, Canada

## Abstract

**Introduction:**

Given the potential malignancy risks associated with computed tomography (CT), some physicians are increasingly advocating for risk disclosure to patients/families. Our goal was to evaluate the practices and attitudes of pediatric emergency medicine (PEM) fellowship program leaders’ regarding CT radiation-risk disclosure.

**Methods:**

We conducted a cross-sectional survey study of the United States and Canadian PEM fellowship directors and associate/assistant directors. We developed a web-based survey using a modified Dillman technique. Primary outcome was the proportion who “almost always” or “most of the time” discussed potential malignancy risks from CT prior to ordering this test.

**Results:**

Of 128 physicians who received the survey, 108 (86%) responded. Of those respondents, 73%, 95% confidence interval (CI) [64–81] reported “almost always” or “most of the time” discussing potential malignancy risks when ordering a CT for infants; proportions for toddlers, school-age children, and teenagers were 72% (95% CI [63–80]), 66% (95% CI [56–75]), and 58% (95% CI [48–67]), respectively (test for trend, p=0.008). Eighty percent reported being “extremely” or “very” comfortable discussing radiation risks. Factors of “high” or “very high” importance in disclosing risks included parent request for a CT not deemed clinically indicated for 94% of respondents, and parent-initiated queries about radiation risks for 79%. If risk disclosure became mandatory, 82% favored verbal discussion over written informed consent.

**Conclusion:**

PEM fellowship program leaders report frequently disclosing potential malignancy risks from CT, with the frequency varying inversely with patient age. Motivating factors for discussions included parental request for a CT deemed clinically unnecessary and parental inquiry about risks.

## INTRODUCTION

There is increasing awareness among the medical community^1^ and the media^2^ of the potential carcinogenic risks from medical radiation. Recent epidemiological studies have added to concerns relating to computed tomography (CT), particularly from exposure during childhood.^3^ As a result, some physicians advocate for disclosure of possible malignancy risks prior to ordering CT imaging in children. CT is a commonly ordered test by pediatric emergency medicine (PEM) physicians,^4^ and children are among the most sensitive to the potential long-term effects of radiation.^5^ Despite this, there has been limited investigation and no firm recommendations for implementing risk disclosure practices for CT imaging of pediatric patients in the emergency department (ED). Further, various approaches towards implementing consent for radiological procedures that expose patients to radiation are currently under debate.^6^ A study of Canadian PEM physicians’ knowledge of potential CT malignancy risks suggested that nearly 70% usually disclose risks to patients and families.^7^ However, there are differences in imaging practices between the United States and Canada,^8^ which may translate into differences in risk-disclosure practice.

The primary objective of this study was to evaluate the frequency with which PEM fellowship program leaders disclose potential malignancy risks from CT to pediatric patients and families. As secondary objectives, we determined physician comfort with risk disclosure and knowledge of malignancy risks, factors deemed of high importance in engaging in risk-disclosure discussions, and respondent preference for disclosure method.

## METHODS

### Study Design and Population

This was a web-based survey of PEM fellowship program directors and associate/assistant directors in the U.S. and Canada from April 10 to June 25, 2015. We compiled an initial list of directors and emails based on data updated and published annually^9^ and information available on the Royal College of Physicians and Surgeons of Canada website (http://www.royalcollege.ca/rcsite/documents/arps/ped-emergency-e). We confirmed names and email addresses for program leadership at each program via the program website, program coordinator, or directly with a program director or associate/assistant director. We excluded those whose email address we were unable to verify or who were no longer in active clinical practice. The University of Pittsburgh Institutional Review Board approved the study.

### Survey Development and Content

We developed survey items in accordance with the methods advocated by Burns et al,^10^ and Dillman.^11^ We derived questions initially from relevant literature^7^, ^12^–^15^ and an expert panel of two emergency physicians, two PEM physicians, and one pediatric radiologist, all of whom had survey and/or content expertise. Questions related to lifetime malignancy risk from CT imaging were based on published estimates.^16^–^19^ The expert panel generated items for the survey until no new items emerged and the final items were agreed upon. We pre- and pilot-tested the initial survey draft with 14 PEM physicians (not involved in fellowship leadership) at three different U.S. academic medical centers. Survey questions were removed or modified in accordance with feedback from all testing phases. In its final form, there were a total of 13 questions (online appendix-survey), and the median time to complete the survey was less than 10 minutes.

**Population Health Research Capsule**

What do we already know about this issue?

Radiation exposure from Computed Tomography may be associated with an increased risk of future cancer, which has raised concerns among patients and clinicians.

What was the research question?

How often do physicians, specifically pediatric EM fellowship program leaders, disclose potential malignancy risks from CT?

What was the major finding of the study?

These physicians report frequently disclosing potential risks, with the frequency inversely proportion to patient age.

How does this improve population health?

Pediatric patients and their families may be increasingly informed of the potential risks of CT prior to undergoing imaging.

The survey included three specific content domains: 1) radiation risk disclosure practice patterns and attitudes; 2) knowledge of radiation exposure from CT imaging; and 3) participant demographics. Respondents were instructed to provide responses assuming they pertained to stable patients for whom there was time for discussion of management options and ability of the parent/guardian to participate in such discussions. We structured questions as either categorical or Likert-scale response types. For all questions, we offered an “other” category in which we solicited a free-text response.

### Survey Administration

We administered the survey through an online survey tool (Qualtrics, Provo, UT) using a modified Dillman’s tailored design method for mail and internet surveys.^11^ An initial e-mail including an introductory letter and link to the survey was sent to eligible participants. Three reminder e-mails were sent at two-week intervals to those who had not yet completed the survey. Each notification described the study, assured confidentiality, and requested participation. Survey responses were de-identified. To incentivize participation, individuals who completed the survey were given the option of being entered into a lottery for a $100 gift card.

### Outcomes

The primary outcome was the proportion of respondents who “almost always” or “most of the time” discussed potential CT malignancy risks prior to ordering CT imaging in stable pediatric patients. Secondary outcomes included the proportion of respondents who felt “extremely” or “very” comfortable discussing potential risks with patients/families, and those factors deemed as being of “very high” or “high” importance in the decision to discuss or not to discuss potential CT risks. We also evaluated the proportion of respondents that favored verbal informed discussion, those educational resources deemed “very” or “somewhat” useful for risk communication, and national campaigns with which respondents were “very” or “highly” familiar. Finally, we examined the proportion that was able to correctly identify estimated relative malignancy risks from a non-contrast head CT. Head CT was chosen given the frequency of its use in the pediatric ED setting.^20^ For questions involving Likert scales, we combined responses into two or three meaningful groups for ease of interpretation.

### Data Analysis

There were 127 PEM fellowship program leaders. Assuming a response rate of 85% based on previous surveys of this population,^21^,^22^ a final sample size of 107 would produce a 95% confidence interval (CI) around the sample proportion of ±9% when the estimated proportion of physicians who at least “most of the time” discussed future potential malignancy risks was 70%.^7^ Partially completed surveys were included, with completed questions used in the analysis. We used proportions with respective 95% CIs to describe the data and the chi-squared test for linear trend to evaluate the relationship between disclosure practices and patient age. We considered a p-value less than 0.05 significant. Stata 12.0, (StataCorp, LP, College Station, TX) was used for statistical analysis.

## RESULTS

### Study Population

We verified information for all 127 fellowship directors and associate/assistant directors from the 78 PEM fellowship programs in North America. One associate program director was excluded for lack of any clinical care responsibilities. Of the 126 eligible physicians, 108 responded to the survey, yielding a response rate of 86%. One hundred and four of the respondents completed the survey in its entirety (96%). Fifty-three percent of respondents were in practice since PEM fellowship for ≤10 years. Respondents represented all regions of the U.S., with 31% from the northeast, 26% from the South, 22% from the Midwest, and 14% from the West. Those from Canada comprised 7% of survey respondents.

### Risk Disclosure Practices and Attitudes

The following proportions of physicians reported discussing potential future malignancy risks “almost always” or “most of the time” for infants, toddlers, school age, and teenage patients, respectively: 73% (95% CI [64–81]), 72% (95% CI [63–80]), 66% (95% CI [56–75]), 58% (95% CI [48–67]), (chi- squared test for linear trend, p=0.008) (Table 1).

Eighty percent of physicians reported feeling “extremely” or “very” comfortable discussing the potential future malignancy risks from CT with parents/guardians; 17% reported feeling “somewhat” comfortable; and 4% reported feeling “a little” or “not at all” comfortable. Of the 108 respondents, 102 (94%) indicated that family request for a CT not deemed to be clinically indicated was of “very high” or “high” importance in their decision to discuss the potential malignancy risks associated with CT (Table 2).

Direct patient/family request for risk information was of “very high” or “high” importance in risk disclosure for 79% of respondents. Sixty-one percent responded that medico-legal implications for not discussing risks were of “very low” or “low” importance. Regarding factors influencing the decision not to discuss the potential malignancy risks from CT, 29% reported that constraints of time pressure and 26% concern that the child’s health would be compromised due to refusal were of “very high” or “high” importance (Table 3).

Survey respondents were asked how they thought risk disclosure should be performed if disclosure became the standard of care. Of the 104 respondents to this question, 40% endorsed verbal discussion without documentation in the medical record, 42% endorsed verbal discussion with documentation in the medical record, and 17% favored written informed consent.

### Physician Knowledge of Radiation Risks

When asked about current estimates^16^–^19^ of the potential increase in lifetime cancer mortality from head CT imaging, one physician responded there was no risk. For the risk to a 5–10 year-old child receiving a head CT compared to an adult, 29% knew the risk was approximately double and 55% thought it was five times greater than for an adult (Table 4).

Proportions of respondents that selected each of the proposed educational tools to assist with communication of risks and benefits from diagnostic imaging as potentially “very” or “somewhat” useful were as follows: online lecture/educational webinar (85%); smartphone app/web-based interactive tool (83%); automated feature of the electronic medical record when ordering a CT (75%); in-person lecture or workshop (68%); and pocket card or short booklet (66%).

Finally, physicians were asked their familiarity with imaging utilization and radiation awareness and safety campaigns and principles. Of the 104 respondents, 59 (57%) were “very” or “highly” familiar with the ALARA (as low as reasonably achievable) principle; 35 (34%) with the Image Gently Campaign; 25 (24%) with the Choosing Wisely® campaign; 22 (22%) with the Image Wisely campaign; and, 19 (18%) with the American College of Radiology Appropriateness Criteria®. Nearly all respondents (96.2%) reported that technical settings on CTs were adjusted for pediatric patients, and four (3.8%) were unsure.

## DISCUSSION

Among PEM fellowship program leaders in North America, the majority reported discussing potential future malignancy risks routinely with parents/guardians. Disclosure frequencies significantly decreased with increasing age of the child, and most physicians reported feeling comfortable with these discussions. The most important motivating factors for initiating risk discussions were family request for a CT that the physician felt was not clinically indicated and direct patient/family request for more information about risk. Most endorsed a verbal process for disclosing potential CT malignancy risks.

Previous studies of physician disclosure of CT malignancy risks have focused on general emergency physicians^12^,^13^,^23^ who primarily care for adult patients,^24^ radiologists,^25^ and pediatric surgeons.^26^ These studies show only a minority (9%–37%) of physicians disclose potential malignancy risks to patients and families. In contrast, a study of Canadian PEM physicians found the majority (69%) reported disclosing risks most or all of the time prior to CT. These findings and ours support a higher rate of risk disclosure among PEM academic physicians. This may reflect greater attention from the medical community^1^,^5^ as well as from the media,^2^ toward highlighting radiation risks in children. Further, our study also suggests a patient age-related trend in risk disclosure practice, consistent with PEM physician awareness of the widely accepted inverse relationship between age of exposure and malignancy risk.^16^

Most respondents knew that the estimated malignancy risk from CT for a child is greater than that for an adult patient, although only a minority of respondents selected the correct relative increase in risk. Many in fact overestimated the relative increased risk. A previous systematic review including seven studies investigating physician awareness of radiation risks found that only an average of 54% believed that ionizing radiation increased the risk of developing cancer.^27^ In our study, all but one respondent believed there was a risk. This may reflect an increase in awareness by PEM physicians, specifically in academic medicine. Publicity surrounding the ALARA principle, as well as high-profile scientific studies,^3^ may have contributed to these findings. Nonetheless, most physicians in our study did advocate for resources to assist with risk-disclosure practices in the ED, in particular an online educational lecture or webinar and a smartphone or web-based interactive tool. This suggests a continued need for education and support for physicians to effectively engage in radiation-risk discussions with patients and families. Interdepartmental collaboration between PEM physicians and radiologists for a consistent and informed approach will be an important element in developing such tools. Furthermore, the majority of respondents were unfamiliar with many of the campaigns designed to increase radiation-risk awareness and imaging appropriateness, indicating organizations need to improve the scope of their imaging awareness campaigns to better include more of the PEM community.

We found that for nearly all respondents, the decision to disclose the potential malignancy risks from CT was strongly influenced by parent/guardian request for a CT that was not deemed clinically indicated. This may be one strategy physicians use to dissuade parents/guardians from requesting unnecessary imaging. This approach relies on “anticipated regret;” that is, it aims to influence a parent’s/guardian’s decision to have their child exposed to the radiation from CT.^28^ It assumes the parent/guardian will no longer request the CT after considering their future regret if the CT is normal but their child develops cancer at some future date. Physicians also identified that initiation of discussion was often prompted by patient/family requests for more information, which reinforces that parents are increasingly aware of possible risks due to media coverage on this topic.^2^ Parents may want to be informed about possible risks before undergoing CT imaging, as demonstrated by one study in which 90% of parents surveyed reported a preference for disclosure.^29^ Most of the potential barriers to risk disclosure proposed in our survey were only identified by a minority of physicians as important factors dissuading them from radiation-risk discussions. More work is needed to further explore facilitators and barriers to radiation-risk disclosure in the pediatric ED in order to promote consistent and effective communication strategies.

To date, risk disclosure for CT imaging has been a matter of debate in the medical community, which is in contrast to other procedures that carry similar and even lower risks.^30^ Consequently, some contend that CT imaging should be subject to written informed consent.^6^,^31^ However, the lack of consensus and certainty of radiation-risk estimates contributes to the argument against a formal, informed consent process. As a result, others advocate for an informed or shared decision-making process,^32^–^33^ which acknowledges the uncertainty in the precise level of risk but accepts that there is likely some small risk. In a commentary published in *Pediatric Radiology*, two steering committee members of the Image Gently campaign advocated that “educational materials be provided to every parent or patient prior to the performance of every CT scan as part of medical safety and practice quality improvement and that receipt of this information be documented…in the electronic medical record.”^33^ Radiologists and PEM physicians will need to collaborate at the hospital, regional, and national level to determine the optimal way to provide information regarding radiation risks from CT to parents/guardians. Additionally, future studies should evaluate the manner in which CT risk disclosure should occur as well as the effects of implementing a standardized consent process on CT utilization rates, parent/guardian satisfaction, and patient outcomes.

## LIMITATIONS

There are limitations to our study. Our study was limited to PEM fellowship program leaders, and thus our data are not generalizable to all PEM physicians. However, practices and attitudes from this physician group may provide information regarding PEM physicians in academic centers, and it is often these centers that shape the direction of PEM in a variety of practice settings.^34^ Further, program directors lead the education of future PEM physicians, who go on to practice PEM in community and academic sites. Nonetheless, further work regarding a broader sample of PEM physicians is needed.

In addition, in some cases there were multiple respondents from a single institution; therefore, some of the responses may not be independent of each other if there is teaching consistency within the program. As with all survey studies, ours is subject to selection bias, in that those who do engage in radiation-risk discussions with parents/guardians may be more willing to complete the survey. However, given the relatively high response rate, this is unlikely to substantively affect our results. Our data indicate what physicians report doing, which may not reflect actual practices. In addition, it is possible that some responses were influenced by social desirability^35^ and resulted in physicians reporting assumed “ideal” practice. These factors may have resulted in an overestimation of disclosure frequencies.

## CONCLUSION

Our study indicates that PEM fellowship program leaders report commonly discussing potential malignancy risks with patients’ parents/guardians, with the frequency increasing with younger patient age. Radiation risk disclosure is often driven reactive to parent/guardian requests. These physicians are aware of the increased CT radiation risk; however, they are in need of more resources to better communicate these risks, and most support a verbal strategy for mandatory risk communication. These data provide information for future work to standardize and optimize the manner in which CT radiation risks are disclosed to patients and families in the ED.

## Supplementary Information



## Figures and Tables

**Table 1:** Frequency of physician disclosure of potential malignancy risk from computed tomography, by patient age group (N=108).

Age group	Almost always	Most of the time	Sometimes	Not very often	Almost never
Infants, n (%)	41 (38)	38 (35)	24 (22)	1 (1)	4 (4)
Toddlers, n (%)	40 (37)	38 (35)	24 (22)	3 (3)	3 (3)
School-age, n (%)	37 (34)	34 (32)	29 (27)	5 (5)	3 (3)
Teenagers, n (%)	30 (28)	32 (30)	32 (30)	11 (10)	3 (3)

**Table 2:** Factors influencing physician decision to discuss potential malignancy risks from computed tomography with parents/guardians (N=108).

Factor	Very high importance	High importance	Moderate importance	Low importance	Very low importance
The patient/family is requesting the CT but I do not think it is clinically indicated n (%)	69(64)	33(30)	4 (4)	1 (1)	1 (1)
Patient/family directly asks me for more information, n (%)	50 (46)	36 (33)	16 (15)	4 (4)	2 (2)
It is my duty to let patients/families know about the potential risks and benefits of any test, n (%)	33 (31)	41 (38)	26 (24)	6 (6)	2 (2)
Patients/families often worry about the potential risks, even if they do not ask, n (%)	23 (21)	41 (38)	30 (37)	9 (8)	5 (5)
There may be medico-legal implications if I do not discuss the risk, n (%)	3 (3)	8 (7)	31 (28)	42 (39)	24 (22)

*CT,* computed tomography.

**Table 3:** Factors influencing physician decision NOT to discuss potential malignancy risks from computed tomography with parents/guardians (N=106).

Factor	Very high importance	High importance	Moderate importance	Low importance	Very low importance
Time pressure, n (%)	3 (3)	27 (26)	30 (28)	30 (28)	16 (15)
Concern that the patient’s health will be compromised due to refusal, n (%)	8 (8)	19 (18)	22 (21)	42 (40)	15 (14)
Concern that patients/families will refuse the CT and/or ask for alternative tests/strategies not easily available, n (%)	6 (6)	9 (9)	29 (27)	44 (42)	18 (17)
Most patients/families will not understand the complexities of these discussions, n (%)	1 (1)	9 (9)	23 (22)	45 (43)	28 (26)
Discussion is not necessary because I as a physician have considered the balance of benefit and risk, n (%)	1 (1)	9 (9)	18 (17)	36 (34)	42 (40)
Discussion is not relevant because there is a lack of consensus on the level of risk, n (%)	2 (2)	5 (5)	18 (17)	45 (43)	36 (34)
Discussion is not relevant for children with reduced life expectancy, n (%)	1 (1)	3 (3)	17 (16)	37 (35)	48 (45)
Lack of confidence in my knowledge of the potentail risk, n (%)	0 (0)	2 (2)	17 (16)	49 (46)	38 (36)

*CT*, computed tomography.

**Table 4:** Physician knowledge of potential increase in lifetime cancer-mortality estimate associated with a single head computed tomography in an adult and pediatric patient.

Risk (N=104)	n (%)
Adult patient (30–50 years-old)	
1 in 100	1 (1)
1 in 1000	17 (16)
1 in 10,000*	38 (37)
1 in 100,000	15 (14)
1 in 1,000,000	4 (4)
Don’t know	28 (27)
There is no risk	1 (1)
Pediatric patient (5–10 years-old)^	
1/5 the risk	0 (0)
1/2 the risk	0 (0)
Similar to adult risk	2 (2)
2 times the risk*	30 (29)
5 times the risk	57 (55)
Don’t know	15 (14)

* Correct Response,

^ Assumes appropriate adjustments to technical settings.
