# Supplementary material for: Computed Tomography Risk Disclosure in the Emergency Department: A Survey of Pediatric Emergency Medicine Fellowship Program Leaders
Source: West J Emerg Med. 2018 Jun 4;19(4):715–21. doi: 10.5811/westjem.2018.4.36895 (PMC6040898; doi:10.5811/westjem.2018.4.36895)
Supplement: Supplementary file 1 [file wjem-19-715-s001.pdf]

Default Question Block

Welcome to the RRAD Survey- Radiation Risks: Attitudes on Disclosures

We thank you very much in advance for completing our survey!

Note: *"Stable"* patient is used in this survey to refer to patients in the emergency department where there is time for discussion of management options and ability of the parent/guardian to participate in such discussions.

1. For *stable* patients, how comfortable do you feel discussing the potential future malignancy risks associated with CT with parents/guardians?

Extremely comfortable	Very comfortable	Somewhat comfortable	A little comfortable	Not at all comfortable
○	○	○	○	○

2. When ordering a CT for a *stable* patient, in general, how often do you discuss the potential future malignancy risk from ionizing radiation with the parent or guardian?

Please provide a separate response for each patient age.

	Almost always	Most of the time	Sometimes	Not very often	Almost never
Infants	○	○	○	○	○
Toddlers	○	○	○	○	○
School-age children	○	○	○	○	○
Teenagers	○	○	○	○	○

Tell us if you have other comments:

3. When you DO discuss the potential malignancy risk associated with CT (for a *stable* patient), how important are the following in your decision to discuss the risk?

	Very low importance	Low importance	Moderate importance	High importance	Very high importance
Parent/guardian directly asks me for more information	○	○	○	○	○
It is my duty to let parents/guardians know about the potential risks and benefits of any test	○	○	○	○	○
Parents/guardians often worry about the potential risks	○	○	○	○	○
There may be medico-legal implications if I do not discuss the risk	○	○	○	○	○
The parent/guardian is requesting the CT but I do not think it is clinically indicated	○	○	○	○	○

Tell us if there are other factors important in your decision to discuss the potential malignancy risk associated with CT:

4. When you DO NOT discuss the potential malignancy risks associated with a CT (for a *stable* patient), how important are the following in your decision NOT to discuss the risk?

	Very low importance	Low importance	Moderate importance	High importance	Very high importance
Time pressure	○	○	○	○	○
Concern that patients/guardians will refuse the CT and/or ask for alternative tests/strategies that are not easily available	○	○	○	○	○
Discussion is not relevant because there is a lack of consensus of the level of risk	○	○	○	○	○
Lack of confidence in my knowledge of the potential risk	○	○	○	○	○
Most parents/guardians will not understand the complexities of these discussions	○	○	○	○	○
Discussion is not necessary because I as a physician have already considered the balance of benefit and risk	○	○	○	○	○
Concern that the child's health will be compromised due to refusal	○	○	○	○	○
The discussion of risk is not a relevant for children with reduced life expectancy	○	○	○	○	○

Tell us if there are other factors important in your decision NOT to discuss the potential malignancy risk from CT:

5. If malignancy risk disclosure to parents/guardians became the standard of care for medical imaging with radiation exposure, how do you think this should be performed?

- Verbal informed discussion without requiring documentation in the medical record *(i.e. conversation between clinician and patient/family discussing potential risks and benefits, including limitations of our knowledge)*
- Verbal informed discussion with documentation in the medical record
- Written informed consent *(signed document acknowledging awareness of potential risks and benefits)*
- Other (please specify):

6. How useful do you think the following education tools would be in providing you with information on risks/benefits from diagnostic imaging so that you can communicate better about these issues?

	Not at all useful	Rarely useful	Somewhat useful	Very useful
Online lecture/educational webinar	○	○	○	○
In-person lecture/workshop	○	○	○	○
Smartphone app/web-based interactive tool	○	○	○	○
Automated feature of the electronic medical record when ordering a CT	○	○	○	○
Pocket card/short booklet	○	○	○	○

Tell us if there are other education tools that would be effective for you:

7. Consider an adult patient (30-50 yrs old) who undergoes a head CT for trauma. What is the current estimate of the potential increase in lifetime cancer mortality associated with this examination?

It is acknowledged that the estimation of potential risk associated with CT examinations is very challenging due to local variability in doses and the many complexities and limitations of CT dosimetry and risk model methodologies. Given these limitations, please select the answer that you feel is most correct to the nearest order of magnitude.

- 1 in 100
- 1 in 1,000
- 1 in 10,000
- 1 in 100,000
- 1 in 1,000,000
- There is no risk
- I do not know

8. Now consider a 5-10 year old child who undergoes a head CT. Appropriate adjustments to the technical settings are made and the effective dose is similar to that of the scan performed on the adult patient.

How does their potential increase in lifetime cancer mortality risk compare to the adult patient?

- Approximately one fifth of the risk (0.2 x adult risk)
- Approximately half the risk (0.5 x adult risk)
- Similar to that of the 30-50 year-old adult
- Approximately double the risk (2 x adult risk)
- Approximately five times the risk (5 x adult risk)
- I do not know

9. Are the technical settings on the CTs obtained through your ED adjusted for pediatric patients?

- Yes
- No
- I am unsure
- Other (please specify):

10. To what degree are you familiar with the following concepts and campaigns?

	Highly familar	Very familiar	Somewhat familiar	A little familiar	Not at all familiar
ALARA (As Low As Reasonably Achievable)					
Image Gently Campaign					
Image Wisely Campaign					

Choosing Wisely Campaign	◯	◯	◯	◯	◯
American College of Radiology (ACR) Appropriateness Criteria	◯	◯	◯	◯	◯

11. In general, how often do you consider a patient's prior radiation exposures from imaging when deciding on appropriate imaging tests for the current ED visit?

Almost always	Most of the time	Sometimes	Not very often	Almost never
◯	◯	◯	◯	◯

Please tell us if you have additional comments regarding prior radiation exposures from imaging:

12. How many years have you been in practice since completing pediatric emergency medicine (PEM) fellowship training?

- ◯ Less than 5 years
- ◯ 5 -10 years
- ◯ 11-15 years
- ◯ 16-20 years
- ◯ More than 20 years
- ◯ I am not PEM fellowship trained

12a. How many years have you been in practice since completing residency training?

- ◯ Less than 5 years
- ◯ 5-10 years
- ◯ 11-15 years
- ◯ 16-20 years
- ◯ More than 20 years

13. Do you primarily practice in the United States or Canada?

- ◯ United States
- ◯

Canada

13a. In which state is the emergency department in which you primarily work located?

13a. In which Canadian province is the emergency department in which you primarily work located?

14. Please provide any additional comments you have regarding this topic:

15. If you would like to be entered into a raffle to receive $100, please provide an email address below.

For educational materials and awareness opportunities regarding radiation from medical imaging, please visit:

http://www.imagegently.org
